# Reviews

**Published:** 1923-01

**Authors:** 


### REVIEWS OF BOOKS.

**LABORATORY MANUAL OF BIOLOGICAL CHEMISTRY.** By Professor OTTO FOLIN, of Harvard Medical School. Pp. 300. (Appleton & Co., New York and London; 12s. 6d.)

The third edition of this well-known work will be welcome in every medical laboratory. In recent years biological chemistry has made such remarkable advances that a book, absolutely and concisely up-to-date, and selecting from the mass of literature just those tests which are really reliable, is an absolute necessity. In his manual Professor Folin has provided exactly the work required. Also it must be remembered that the author has himself originated and perfected a remarkable number of modern biological methods applicable to the average clinical laboratory, and it is obvious that knowledge of such subtle technique is far better learned from the inventor's own descriptions.

The most noteworthy chapters are those devoted to chemical analysis of the blood. This particular branch of laboratory work is daily receiving more attention from clinicians, and in connection with it Folin's name is deservedly famous. The subject is most inadequately dealt with in the majority of works commonly read by the British medical student, and for these pages alone the manual would deserve its place in our libraries.

Of the appearance of the book, one remarks that only the right-hand sheets carry type, the left-hand in every case being quite blank, a format which not only facilitates reading, but provides also valuable space for personal laboratory notes, &c. In this connection the writer has only one criticism to make —and that a small one. For rapidity of reference it would have been helpful to place, at the top centre of each page, the name of the fluid or tissue to which that page referred. At present it is not always easy without reading well into the paragraphs or referring back to the beginning of the chapter to know exactly which section is being dealt with. The time factor is important in laboratory work, and heading up of the pages in this manner might, we think, often save some valuable moments. Otherwise the general production is excellent and the quality of both paper and printing leave nothing to be desired.

---

**LE PROBLÈME DU CANCER.** Par WILLIAM SEAMAN BAINBRIDGE, A.M., Sc.D., M.D., Professeur de Chirurgie, New York Polyclinic Medical School and Hospital; Chirurgien et Secrétaire de Recherches au New York Skin and Cancer Hospital. Traduit de l'Anglais par le Dr. Hertoghe d'Anvers, Membre titulaire de l'Academic Royale de Belgique, d'après la première édition de la Macmillan Company de New York. Presse de Trois Rois, Louvain Universitaire, 1922. Pp. 484 + xiv.

This is a translation of the English edition which appeared in 1915, and has been printed by the Press of Louvain University, the first work to appear by that press since its buildings were destroyed by the Germans in 1914.

This book since its original English edition has been revised, brought up to date and contains much that is new.

The book is divided into fourteen parts, each part being complete in itself. The first part deals with the historical side of cancer and the various institutes and laboratories used for the study of that disease. Other parts deal with the etiology, prophylaxis, treatment, &c. Emphasis is laid on the early diagnosis, careful and efficient surgical treatment combined with X-rays and radium.

Part IX deals with the anti-cancerous medications; they are many and various, and in these the author's belief is negligible.

Part XIII deals with the Institutions destined for incurable cases both in America and Europe. The last part deals with the education of the public in regard to the early signs and symptoms of the disease and the seeking of advice in its early phases.

This book deals with the problems surrounding cancer in a comprehensive, interesting and practical way and should be of great value to all practitioners of medicine.

---

**PREVENTIVE MEDICINE AND HYGIENE.** By MILTON J. ROSENAU. 4th ed. 1921. (D. Appleton & Co.)

The fourth edition of this well-known and deservedly popular text-book, in addition to being largely rewritten, contains much new matter relative to the various branches of public health. As the author aptly remarks, the whole ideal of preventive medicine is "to build towards departments dealing with health rather than departments dealing with disease." One of the weaknesses of our own public health departments is the comparatively small attention paid to the educative aspect of the work—*i.e.*, the diffusion of knowledge among the people by the various propaganda methods—many of which are in common use in the United States. In discussing the relative values of public health work the views of Chapin and Schneider are quoted to show the great relative importance of child hygiene, including school inspection, and infant welfare, which is placed second to the control of the communicable diseases. The author's sound judgment, combined with a legitimate amount of optimism is seen on p. 176, where it is stated that "although there is some doubt concerning the exact mode of transmission and the portal of entry that the tubercle bacillus usually takes, we have sufficient knowledge to guide our preventive measures with every assurance of success." He further states that "a wholesome regard for the infection is useful and helpful in preventive medicine, but an hysterical fear of tuberculosis is quite as unwarranted as a total disregard for the infection."

A good account is given of vitamines and of the deficiency diseases, including "war œdema." The importance of thorough heating and of preserving only fresh and clean food are emphasised in the prevention of botulism. Among the diseases that are described for the first time in this book are Leishmaniasis, deer-fly fever and epidemic encephalitis. In the section dealing with military hygiene the experience gained in the Great War has been utilised. With regard to vaccination and its benefits the statement on p. 25 is specially applicable at the present time : " To remain unvaccinated is selfish in that by doing so a person steals a certain measure of protection from the community on account of the barrier of vaccinated persons around him."

To all students of hygiene in its most comprehensive sense, as well as to public health administrators, this volume may be warmly commended.

---

AN INDEX OF PROGNOSIS AND END RESULTS OF TREATMENT. Edited by A. RENDLE SHORT, M.D., F.R.C.S. 3rd ed. 1922. Pp. 594. (J. Wright & Sons; 42s. net.)

This publication has long ago reached general acceptance as the standard English reference book on the subject it deals with. More even than most other medical text-books, it requires constant revision to keep abreast of current progress, and the best modern practice ; and this revision it effectively receives at the hands of the editor and his band of contributors. Even where no substantial change has taken place for a few years in the treatment of a given malady—for instance, in the operative treatment of cancer of the breast—yet the lapse of years means altered statistics, not only by reason of the greater length of time over which operation cases can be watched, but also by reason of the steady improvement of the rank and file of the profession in diagnosis and of the gradual education of the general public as to the importance of early advice and treatment. To the practitioner-in-the-street hitherto unacquainted with this volume, it may appear to be one mainly useful to surgeons, or at any rate to specialists. It is not so in reality ; the book is a rich mine of information as to the best methods of treatment, because it gives the most authoritative figures as to the results of contrasted methods of treatment, and thus really helps enormously to decide which of two or more alternatives ought to be advised for any particular case. We recommend it as a profitable exercise for any medical man to recall a dozen of the most interesting medical or surgical cases he has seen in the previous month, and then to look up in this index what there is to be said for and against the lines of treatment that have been chosen. All statistics, to be of any use, need to be studied by informed readers ; problems of prognosis are no exception to this rule, and lay readers would be well advised not to look up in this book the prognosis of such maladies as they or their friends may chance to suffer from.

---

A CLINICAL TREATISE ON DIABETES MELLITUS. By MARCEL LABBÉ, M.D. Translated, revised and edited by CHARLES GREENE CUMSTON, M.D. (Wm. Heinemann, Medical Books, Ltd.; 18s. net.)

This is a valuable study of diabetes by an experienced and well-known authority. Diabetes is classified into two groups, according to whether denutrition is or is not a feature ; the former group represents those cases for which the label diabetes mellitus is reserved in this country. Biochemical studies are lucidly described and useful chapters upon the clinical phenomena of glycosuria and its complications are included.

MEDICAL PRACTICE IN OTAGO AND SOUTHLAND IN THE EARLY DAYS. By ROBERT FULTON, M.D. ("Otago Daily Times," Dunedin, N.Z.)

The history of a settlement of a new area of the world is always full of interest, and none is more so than that of the doctors who played their part amongst the pioneers, before life had assumed the fixed order and routine of the country they had left. As a rule, the life of the first settlers in a new country is so strenuous that but little material survives for the compilation of such a history. There are, however, not infrequently still in existence private letters and diaries which are full of those small personal incidents and anecdotes which form a basis for such a historial survey, not to mention the memories and reminiscences of the aged survivors from the early days. Dr. Robert Fulton, son of one of the original settlers, and one of the founders of the Otago Early Settlers' Association, has managed to collect much material of this kind concerning those who practised medicine in that part of New Zealand in the middle of last century. The earliest pioneer of medicine there seems to have been Dr. Joseph Crocone, who, having lost all his belongings in a wreck, settled among the whalers in 1838. In 1847 the first ship of settlers arrived, bringing with them Dr. Henry Manning, who was the first to practise in the Otago settlement. Later on, the rush for gold brought a large influx of all sorts and conditions of men into the district. With them came a succesion of surgeons and physicians. One of these may be picked out for special mention—Mr. John Eccles, who had been a fellow-student and friend of Paget (Sir James) at St. Bartholomew's Hospital. When a change was made in the constitution of the Royal College of Surgeons, Eccles was elected a Fellow. After several years of successful practice at Tonbridge, the lure of New Zealand led him to seek his fortune there. He reached Dunedin after a voyage of 101 days, but without his wife, for she had died a few days before landing from the exhaustion of sea-sickness. Dr. Fulton appropriately dedicates his book to the memory of the wives of the doctors who shared the trials and hardships of their husbands. In these days of quick travel, telegraphs and easy communications, it is difficult for us to realise the sacrifices of those who made the great adventure to build a home in what was veritably another world. The author has been able to collect a large number of photographs of these pioneer practitioners, together with a number of views showing the early aspect of the town and its rapid development. When it is remembered that there are 304 post-octavo pages in double columns of small type, the amount of material that Dr. Fulton has managed to assemble will be appreciated. It is a worthy record, well worth the labour that he has expended upon it.

---

MY MOORLAND PATIENTS. By R. G. W. BISHOP, M.D. (John Murray; 12s. net.)

The restless hurry of life, and the advent of all those inventions of science which have abolished distance, together with the introduction of a general system of elementary education, are fast bringing us all to a dull level of uniformity. "The old order changeth," and there are now but few localities where the habits of thought, the speech, and the homely wisdom of our forefathers still survive. These too, alas! are fast dying out, and before another century is past they will have vanished for ever. Happily here and there a chronicler has appeared before it is too late to give a vivid picture of the human types which may still be met with in the hills and dales of the Pennines or the Northern Fells. No one is in a better position to undertake this task than is the country doctor, who is brought into such close and peculiar intimacy with the characters and prejudices of those who seek his aid. He, above all other men, has "an eye

That hath kept watch o'er man's mortality."

In this he possesses opportunities greater than those of the country parson, but the combination of a keen sense of literary style, a ready sympathy and a rich humour with these opportunities is by no means common. Dr. Bishop possessed all these in no small degree. Practising for many years in the moorland districts of Derbyshire and Yorkshire, he became the victim of a slowly progressing malady. During the last year of his life he solaced himself by writing his reminiscences, which it had been his intention to publish anonymously. But before this could be accomplished Death had summoned him, and there was therefore no valid reason why his name should not be attached to his work. His descriptions are so true to life and to type, and are delineated with so happy and delicate a touch that, to those of us the part or the whole of whose lives have been passed among the moorland folk, there is a wistful pathos in the stories which he tells of his patients. Dr. Bishop was essentially a lover of the country, keenly interested in the history, archæology and dialect of his neighbourhood, and above all else an ardent sportsman. More than all this, he had a kindly feeling with the foibles of his neighbours that made him welcome in the homes of every one in his widely-scattered district. Though written in lighter vein, "My Moorland Patients" bears comparison with Canon Atkinson's "Forty Years in a Moorland Parish." This is high praise. The photographs of moorland scenery are delightful, and the book is full of fascination.

---

**PSYCHOLOGY AND MENTAL HYGIENE FOR NURSES.** By Mary B. Eyre. Pp. 208. (Macmillan Co., New York.)

The nurse—and, indeed the student—is beset by a multitude of counsellors. Whether there is always a corresponding amount of wisdom is quite another matter. Particularly is this so in respect to the subjects treated of in this little volume. It is so often a case of "much cry and little wool." It is the more gratifying, therefore, to be able to recommend a book such as this, where much information is given in compact yet readable form, and where the practical is not submerged by the theoretical. In this connection it is interesting to note that the author does not regard psychology from a transcendental point of view, but considers that it is "based scientifically upon the study of the antomy and phyisology of the brain." When we came upon this passage, we heaved a sigh of relief, and read on with renewed zest. In addition to the purely psychological part—which is quite up-to-date in that the teaching of the new school of psychology is considered—the practical applications of the results obtained are clearly demonstrated, as, for example, in regard to the psychology of occupation and of nursing in particular, and in a discussion of mental hygiene in public health nursing. A section is devoted to the Binet-Simon measuring scale for intelligence.

It is a little difficult to be sure of errors in spelling when one is considering books printed in the United States—but "philogenetic" is surely more correctly spelt "phylogenetic"; while "autogenetic" does not mean "development of the individual." This does not seem to be a printer's error, as it is several times repeated. The correct word is "ontogenetic." This is but a small matter in this handy volume. It is well printed, and has a glossary, and also a good index.

---

### THE VISITING NURSES' DIRECTORY

(The Scientific Press, Ltd.; post free, 10½d. net.)

This little book, which contains the names, addresses, and qualifications of nurses willing to undertake daily nursing, should be exceedingly useful, not only to doctors, but also to those who may need the services of a nurse, but do not wish her to be resident. The lists are conveniently compiled under districts in London, the Provinces, Scotland and Wales, and a blank page is provided opposite each list for recording, for future reference, additional information concerning the nurse engaged. The very moderate price of this compact little directory is an additional recommendation.

---

### C.M.B. EXAMINATION QUESTIONS AND MODEL ANSWERS.

A little handbook which will commend itself to all intending candidates for the Central Midwives' Board examinations, as well as to teachers of midwifery, has just been issued by the Scientific Press, 28/29 Southampton Street, Strand, W.C.2, price 1s. 6d. net. It is of convenient pocket size, strongly bound and neatly got up. In it are the question papers of the C.M.B. examinations during the past three years, together with model answers by a certified midwife-teacher, which have appeared from time to time in the pages of The Nursing Mirror. A careful study of these will familiarise the candidate with the answering of questions put in different ways on the same subjects, a method of knowledge-testing that sometimes proves a stumbling-block to those unused to the ordeal of facing written examination papers, but which to a persevering student, leads to a firmer grip on the principles taught. There are also instructions as to the procedure necessary to become a certified midwife, and a complete list of "approved" teachers, also of "approved" schools at which a pupil-midwife may be trained. The rules of the Central Midwives' Board (abridged) are included at the end of the book, as well as other information, thus making it a very useful guide to all seeking training in this important branch of nursing work.

---

The Committee of the Catherine Gladstone Convalescent Home at Mitcham have given the house, equipment and an endowment fund of £20,000 to the London Hospital. The house, which stands in some five acres, has seventy-five beds.

"The Duchess of Albany Ward" at the Royal Waterloo Hospital for Women and Children is to be endowed in memory of her. The Duchess was president of the Ladies' Association of the hospital.

